# Ciclopirox targets cellular bioenergetics and activates ER stress to induce apoptosis in non-small cell lung cancer cells

**DOI:** 10.1186/s12964-022-00847-x

**Published:** 2022-03-24

**Authors:** Junwan Lu, Yujie Li, Shiwei Gong, Jiaxin Wang, Xiaoang Lu, Qiumei Jin, Bin Lu, Qin Chen

**Affiliations:** 1 Protein Quality Control and Diseases Laboratory, School of Laboratory Medicine and Life Sciences, Wenzhou Medical University, Wenzhou, 325035 Zhejiang China; 2 Department of Laboratory Medicine, Wuhan Pulmonary Hospital, Wuhan Institute for Tuberculosis Control, Wuhan, 430030 Hubei China; 3 Department of Biochemistry and Molecular Biology, School of Basic Medical Sciences, Hengyang Medical School, University of South China, Hengyang, 421001 Hunan China; 4 Department of Intensive Care, The First Affiliated Hospital of Wenzhou Medical University, Wenzhou, 325000 Zhejiang China; 5 Present Address: School of Medicine, Jinhua Polytechnic, Jinhua, 321007 China

**Keywords:** Non-small cell lung cancer, Ciclopirox, Epithelial-mesenchymal transition, Cellular bioenergetics, Endoplasmic reticulum stress

## Abstract

**Background:**

Lung cancer remains a major cause of cancer-related mortality throughout the world at present. Repositioning of existing drugs for other diseases is a promising strategy for cancer therapies, which may rapidly advance potentially promising agents into clinical trials and cut down the cost of drug development. Ciclopirox (CPX), an iron chelator commonly used to treat fungal infections, which has recently been shown to have antitumor activity against a variety of cancers including both solid tumors and hematological malignancies *in vitro* and *in vivo*. However, the effect of CPX on non-small cell lung cancer (NSCLC) and the underlying mechanism is still unclear.

**Methods:**

CCK-8, clonal formation test and cell cycle detection were used to observe the effect of inhibitor on the proliferation ability of NSCLC cells. The effects of CPX on the metastasis ability of NSCLC cells were analyzed by Transwell assays. Apoptosis assay was used to observe the level of cells apoptosis. The role of CPX in energy metabolism of NSCLC cells was investigated by reactive oxygen species (ROS) detection, glucose uptake, oxygen consumption rate (OCR) and extracellular acidification rate (ECAR) experiments. Western blot was used to examine the protein changes.

**Results:**

We report that CPX inhibits NSCLC cell migration and invasion abilities through inhibiting the epithelial-mesenchymal transition, impairing cellular bioenergetics, and promoting reactive oxygen species to activate endoplasmic reticulum (ER) stress-induced apoptotic cell death. Moreover, CPX intraperitoneal injection can significantly inhibit NSCLC growth *in vivo* in a xenograft model.

**Conclusions:**

Our study revealed that CPX targets cellular bioenergetics and activates unfolded protein response in ER to drive apoptosis in NSCLC cells, indicating that CPX may be a potential therapeutic drug for the treatment of NSCLC.

**Graphical Abstract:**

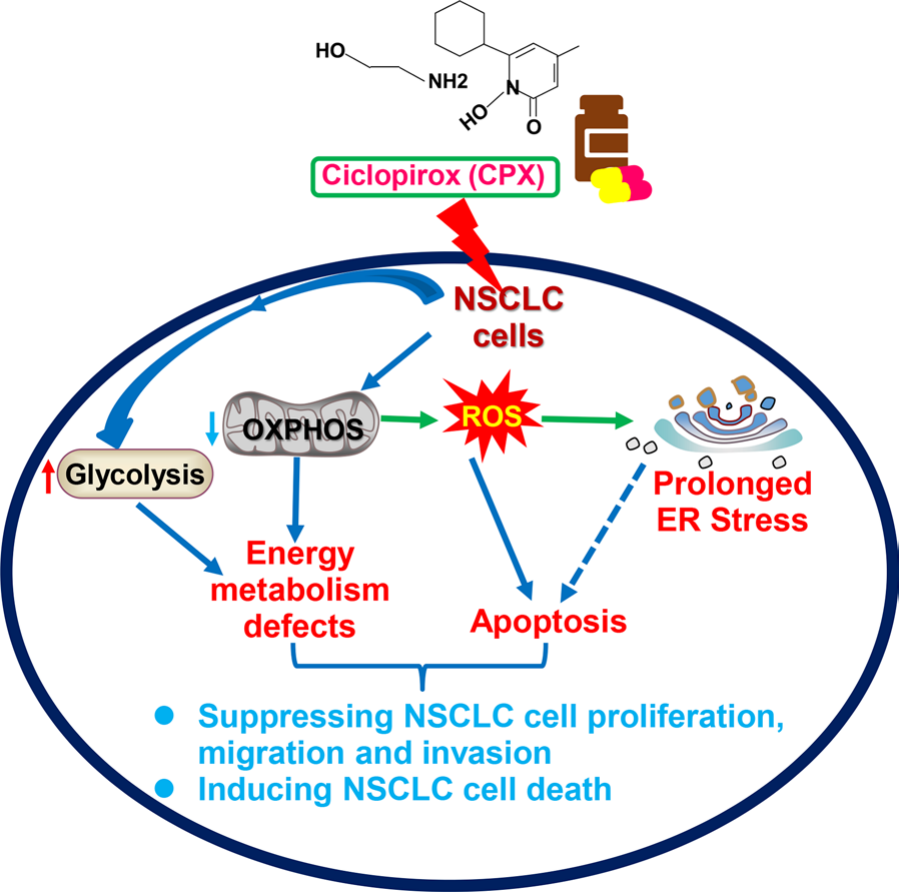

**Video Abstract**

**Supplementary Information:**

The online version contains supplementary material available at 10.1186/s12964-022-00847-x.

## Background

Lung cancer remains a worldwide major public health problem and the leading cause of cancer-related mortality throughout the world, with 1.8 million deaths in 2020 [1]. Lung cancer is mainly divided into two types, small cell lung cancer (SCLC) and non-small cell lung cancer (NSCLC), and NSCLC accounts for 85% of all lung cancers [2–4]. Despite clinical diagnosis and treatment have improved, the 5-year overall survival after diagnosis is still very poor, especially in advanced patients, because of the increased resistance of targeted therapies [1, 5]. Therefore, development of new drugs and therapeutic strategies to improve the treatment efficiency and prognosis of patients with NSCLC becomes more and more urgent.

Ciclopirox (CPX) is an off-patent antifungal agent, which has been documented to be an effective anticancer agent against both hematologic malignancies and solid tumors [6–12]. CPX treatment significantly upregulated genes involved in hypoxia, glycolysis, MTORC1 signaling and immune response related process [13]. Pharmacological and toxicological studies further support that CPX and its derivatives have potential to be re-purposed as anti-cancer drugs [14]. To a great extent, however, the mechanism underlying the anti-cancer activity of CPX remains uncharted, which restricts the further application of CPX and its derivatives in clinical practice.

We previously reported that PERK-dependent endoplasmic reticulum (ER) stress was activated by CPX to induce apoptosis in human colorectal cancer (CRC) cells [7]. ER is mainly responsible for protein synthesis, folding, posttranslational processing, structural maturation and assembly of over a third of cellular proteins important for normal functions of cells [15, 16]. A wide range of cellular stimuli may impair the protein homeostasis in the ER and result in overwhelmingly accumulation of misfolded and/or unfolded proteins in the ER lumen above a critical threshold, therefore activating the unfolded protein response (UPR) to cope with this state referred to as “ER stress” [17, 18]. However, if the UPR of ER (UPR^ER^) fails to re-establish the homeostasis and ER stress cannot be mitigated, the UPR^ER^ would serve as an apoptotic executor that activates cell death signaling cascades and the cells undergoing apoptosis [18–20]. Therefore, ER stress induced apoptosis might be a potential strategy to enhance chemosensitivity.

This study aims to investigate the impact of CPX on regulating NSCLC cell growth, migration and invasion. Herein, we found that CPX treatment resulted in cell cycle arrest at G1 phase in NSCLC cells via regulating the expression of cell cycle-related proteins. In addition, we demonstrated that CPX treatment inhibits NSCLC cell invasion and migration through inhibiting the progression of epithelial-mesenchymal transition (EMT) of NSCLC cell. Interestingly, CPX treatment led to NSCLC cell mitochondrial respiratory dysfunction and aerobic glycolytic function enhancement, thus further inhibiting the growth, invasion and migration of NSCLC cells. Moreover, we found that CPX enhanced reactive oxygen species (ROS) activated ER stress and UPR^ER^ to drive cell death in NSCLC cells. Collectively, our research revealed the roles and mechanisms of CPX in inhibiting NSCLC, which suggests that CPX may act as a potential drug for NSCLC patients.

## Methods

### Reagents

Cell Counting Kit-8 (CCK-8), Total ROS Assay Kit, Crystal violet solution and N-acetyl-L-cysteine (NAC), Z-VAD-FMK and TUNEL Apoptosis Assay Kit (C1091) were purchased from Beyotime Biotechnology (Shanghai, China); Antimycin A, Rotenone, Carbonyl cyanide 4-(trifluoromethoxy)phenylhydrazone (FCCP), Oligomycin, 2-Deoxyglucose (2-DG) and L-glutamine were purchased from Sigma-Aldrich; CPX was from Dibo Chemical Technology (Shanghai, China); Annexin V-FITC/PI Apoptosis Double Dye Detection Kit was obtained from BD Biosciences; the Pierce BCA^TM^ protein assay kit, MitoSOX^TM^ Red and Enhanced chemiluminescence (ECL) were from Thermo Fisher Scientific; Protease inhibitor cocktail was purchased from APExBIO, Houston, TX, USA); DNeasy Blood & Tissue Kit (Cat. No.: 69504) was from QIAGEN.

### Cell culture

H1299 and 95D cell lines were purchased from Chinese Academy of Science Cell Bank (Shanghai, China); 293T and HK2 cells were obtained from Procell Life Science&Technology (Wuhan, China). DMEM, MEM, and RPMI 1640 medium was purchased from Life Technologies; Fetal bovine serum (FBS) was purchased from Excell Bio (Shanghai, China). H1299 cells were cultured in RPMI 1640 medium, 95D and 293T cells were cultured in DMEM, while HK2 cells were maintained in MEM. All culture media were supplemented with 10% FBS, 100 U/mL penicillin and 100 μg/mL streptomycin at 37°C in a 5% CO_2_ cell culture incubator, which were routinely confirmed to be free of mycoplasma in this work. Subconfluent cells (70–80% confluence) were used for all experiments.

### Cell viability and proliferation assay

CCK-8 assay was performed to assess the cell viability and proliferation as described previously [7]. Briefly, H1299, 95D, 293T and HK2 cells (5,000 cells in 100 μl of cell culture  medium per well) were seeded into a 96-well plate and incubated overnight. Then the cells were treated with control (DMSO) or increased CPX concentration (0, 5, 10, 20, 40 μM). After 72 h, the cell viability was determined by CCK-8 assay following the manufacturer's protocol. Cell viability of control cells (DMSO treatment) was set at 100%. For the cell proliferation assay, H1299 and 95D cells (3000 cells in 100 μl of cell culture  medium per well) were seeded into a 96-well plate and incubated overnight. The next day, cells were treated with control (DMSO) and 10, 20 and 40 μM of CPX for 1, 2, 3, or 4 days, and proliferation rates were examined by CCK-8 assay.

### Colony formation assay

Cells (H1299 or 95D cell line) were seeded into a 6-well plate at a density of 1000 cells per well at 37 °C in a 5% CO_2_ cell culture incubator. The cell culture medium was replaced every two days. After incubation for 7–10 days, the RPMI 1640 medium was replaced with that containing 5, 10, 20 μM CPX or DMSO. The cells were cultured to be visible to the colony and stained with 0.5% crystal violet as previously reported [21]. Clusters were evaluated with a light microscopy, and each cluster containing over 50 cells was counted as a colony. The number of colony was counted using ImageJ plus and representative images were photographed. Three separate replicate wells were set for each concentration for determination.

### *In vitro* cell migration and invasion assays

To perform the *in vitro* cell migration assay, the cells (2×10^4^) in RPMI 1640 medium without FBS were inoculated into the upper chamber of the transwell plate and 600 μl of RPMI 1640 medium with 15% FBS in the lower chamber. The plate was incubated in cell culture incubator at 37°C with 5% CO_2_ for 12 h, and then using FBS-free medium containing different concentration of CPX or DMSO to replace the medium in upper chamber. The cells were further incubated for another 48 h, and migrated cell was fixed with methanol for 30 min, followed staining with 0.5% crystal violet solution. Images of five fields in each well are randomly selected and captured by inverted microscope. They were quantified by ImageJ Plus. To evaluate the cell invasion ability, the liquefied Matrigel was diluted with F-12 in a volume ratio of 1:9. To obtain a thin gel layer, the upper chamber of the transwell plate was added with an amount of 40 μl of matrigel mixture, and incubated in the 37℃ incubator for 12 h. Then, 50 μl of warm F-12 medium free of serum was added in each chamber and the matrigel layer was rehydrated in a cell culture with 5% CO_2_ at 37°C for 30 min. And the other steps are the same as those for the cell migration analysis.

### RNA isolation and qPCR analysis

Total RNA was extracted from cultured cells by TRIzol^TM^ reagent (Thermo Fischer Scientific, NY, USA). HiScript II Q RT SuperMix for qPCR (Vazyme, Nanjing, Jiangsu, China) was used for reverse transcription. qPCR was conducted as described previously [22]. *β-ACTIN* acts as an internal control and the primer sequences (*ND1* and *COX 1*) for qPCR are shown in Additional file 1: Table S1.

### Intracellular and mitochondrial ROS assay

The 2’,7’-Dichlorodihydrofluorescein diacetate (DCFH-DA) intracellular ROS probe and MitoSOX^TM^ Red were used to detect intracellular ROS and mitochondrial ROS as previously described.

### Cell cycle distribution analysis and apoptosis assay

H1299 or 95D cells (3 × 10^5^) were inoculated in a 6 cm cell culture dish in a 5% CO_2_ cell culture incubator at 37℃ for 12 h. The next day, cells were incubated in the medium containing DMSO (vehicle control) or CPX (5, 10, 20 µM) for 48 h. Then the cells were harvested and fixed overnight with ice-cold 70% Ethanol at 4 °C. After RNase treatment, they were stained with propidium iodide (PI) for 30 min at 4 °C in the dark. Cell cycle distribution was analyzed by flow cytometry using the BD Accuri™ C6 Flow Cytometer (BD Biosciences).

For apoptosis assay, H1299 and 95D cells (3 × 10^5^) were added in a 6 cm cell culture dish in a 5% CO_2_ cell culture incubator at 37 ℃ for 12 h. The next day, cells were incubated in the medium containing DMSO (vehicle control) or CPX (5, 10, 20 µM) for 48 h. Then cells were harvested and stained with Annexin V-FITC/PI for 20 min. Cell apoptosis was analyzed by flow cytometry using the BD Accuri™ C6 Flow Cytometer (BD Biosciences).

### Mouse xenograft study

Male BALB/c nude mice were housed in a specific pathogen-free facility. H1299 cell suspension (5 × 10^6^ cells per mouse) in 100 μl PBS was injected subcutaneously into the left flank of the 4-week-old nude mice (n = 12). When the average tumor volume was close to 75–100 mm^3^, they were randomly divided into two groups (six for each group). The mice in one group were injected intraperitoneally with 20 mg/kg of CPX, and the other group with 0.9% NaCl only, the injection repeated once a day for 2 weeks. The tumor size (length and width) was measured using a digital caliper every other day to monitor the growth of the tumor. The formula was used to evaluate tumor volume at indicated time points: 1/2 × L × W^2^, with L denoting the longest tumor diameter and W is the shortest. Mouse was weighed every other day. Two weeks later, all of the mice were sacrificed and the tumors were dissected, weighed, and photographed.

### Immunohistochemistry (IHC) staining

IHC staining was performed as described previously [22].

### Measurement of mitochondrial DNA (mtDNA) content

The mtDNA content (copy numbers) was measured as described previously [7]. The primer sequences are shown in Additional file 1: Table S2.

### Western blot

The procedures for performing Western blotting analysis have been described previously [21]. The antibodies used in this study are shown in Additional file 1: Table S3.

### Mitochondrial respiration and glycolysis analysis

Real-time bioenergy state changes in oxygen consumption rate (OCR) and extracellular acidification rate (ECAR) were measured using Seahorse Bioscience XF-96 Extracellular Flux Analyzer (Agilent Technologies). H1299 or 95D cells were inoculated in an XFe96 cell culture plate at a density of 3 × 10^4^ per well and incubated at 37℃ in 5% CO_2_ cell culture incubator for 12 h. The following day, cells were incubated with different amounts of CPX or DMSO (control) for 8 h. Then, cells were washed with XF assay medium and kept in a normal incubator without CO_2_ at 37 °C for 1 h. OCR was measured by loading 1 μM oligomycin, 1 μM FCCP, and 1 μM rotenone and antimycin A in XF assay medium, respectively, into the injection port in the XF-96 sensor cartridge. ECAR was measured by loading XF assay medium containing 10 mM glucose, 1 μM oligomycin, and 100 mM 2-DG, respectively, into the injection port in the XF-e96 sensor cartridge. There are 6 replicates for each experiment.

### Statistical analysis

Data were presented as mean ± SD from at least three independent experiments for all statistical data in this study. Data between the two groups were compared with student t-test. All statistical analyses were performed by GraphPad Prism 7.0 Plus, and *p *< 0.05 was considered significant (* for *p* < 0.05, ** for *p* < 0.01, *** for *p* < 0.001, **** for *p* < 0.0001; ns= not significant).

## Results

### CPX dramatically suppresses NSCLC growth *in vitro*

**Fig. 1:**
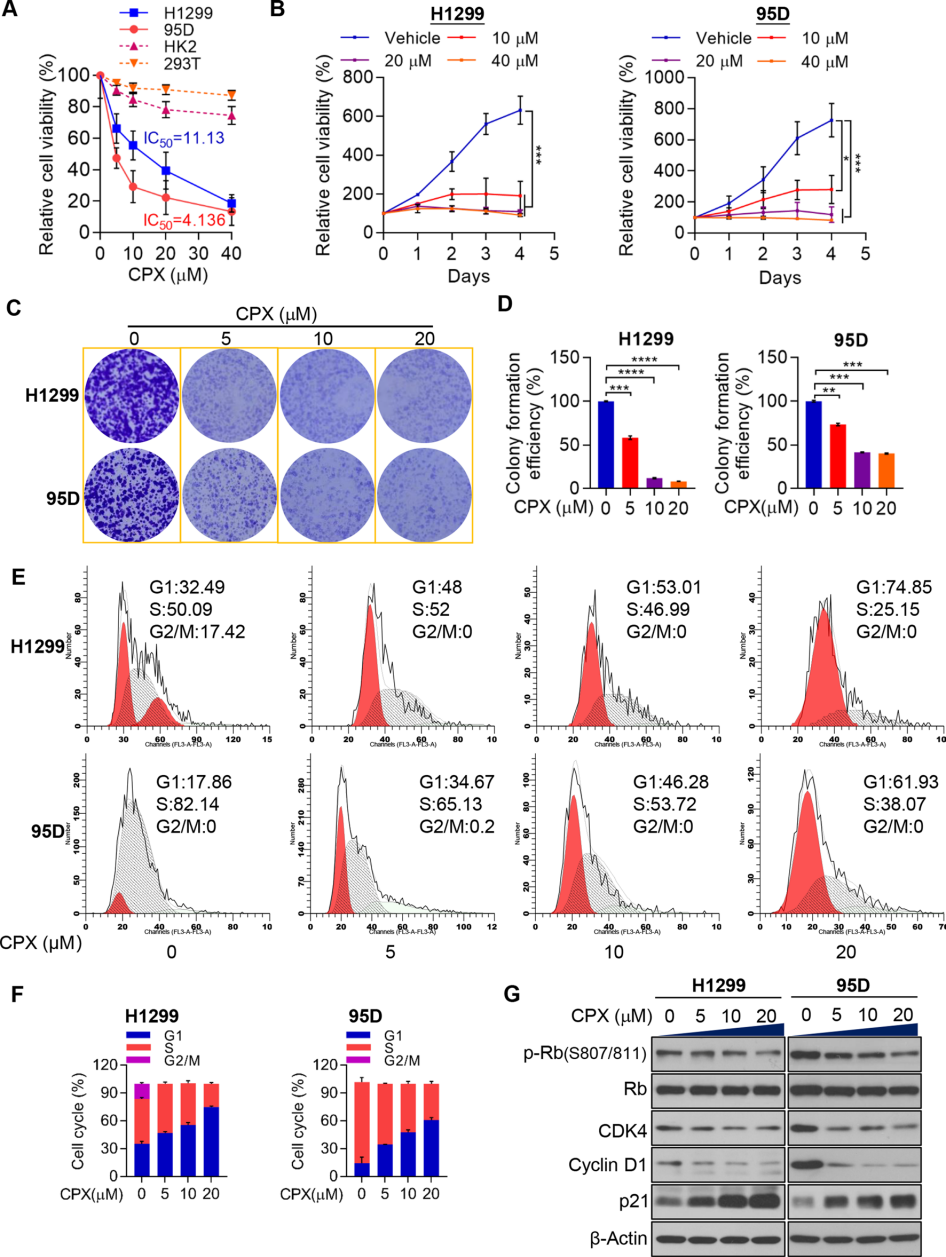
CPX suppresses the growth of NSCLC cells *in vitro*. **A** H1299, 95D, HK2 and 293T cells with the treatment of different concentrations of CPX or vehicle control for 72 h. The relative cell viability for CPX in H1299, 95D, HK2 and 293T cells was determined using CCK-8 assay kits. **B** Cell viability assay of H1299 and 95D cells following treating with various concentrations of CPX (0, 10, 20 and 40 μM) at 1, 2, 3 and 4 days determined by CCK-8 assay. **C** and **D** 1 × 10^3^ H1299 and 95D cells inoculated and cultured in 6-well plates for 5 days, following treating with indicated concentrations of CPX or vehicle control for one week. Representative images showing crystal violet staining of cell colonies (**C**), and colony forming efficiency was determined (**D**). **E and F** The distribution of cell cycle was analyzed by flow cytometry. **G** H1299 and 95D cells treated with various concentrations of CPX as indicated for 48 h. Expression level of cell cycle-related proteins were detected. Data are presented as mean ± SD (n = 3, ns: no significance for indicated comparison. **for *p* < 0.01, ***for *p* < 0.001, ****for *p* < 0.0001)

Previous studies have confirmed that CPX activated PERK-dependent ER stress and thus leading to apoptotic cell death of CRC cells [7]. Similarly, low micromolar concentrations of CPX reduced cell growth and viability in solid tumor cell lines, malignant myeloma, leukemia, and in samples from primary AML patients as well [6]. In the present study, cell proliferation and viability were evaluated to study the anticancer activity of CPX against the NSCLC cells. CPX demonstrated a strong anti-tumor activity in H1299 and 95D cells, which inhibits cell viability in a dose-dependent manner (Fig. 1A, B; Additional file 1: Fig. S1). In contrast, the CPX has only minor effect on 293T and HK2 cells, both of which are non-cancer cell lines (Fig. 1A). Cell proliferation and colony formation were analyzed to further reveal the anti-proliferative activity of CPX. Treatment with CPX (0, 5, 10, and 20 µM) could reduce the clonogenic ability of the NSCLC cells significantly (Fig. 1C, D). Moreover, we found that CPX significantly arrested both H1299 and 95D cells in G1 phase (Fig. 1E, F; Additional file 1: Fig. S2). The cell cycle-related protein expression levels in NSCLC cells with the treatment of CPX were detected to understand the molecular mechanism of CPX in suppressing NSCLC growth. After CPX treatment for 48 h, Cyclin D1 and CDK4 in NSCLC cells were significantly decreased (Fig. 1G). As expected, p-Rb/Rb protein levels were significantly reduced, while p21 was significantly increased (Fig. 1G). These findings suggested that the anticancer activity of CPX for NSCLC was at least partially via arresting the cell cycle.

### CPX suppresses NSCLC cell migration and invasion through inhibiting epithelial-mesenchymal transition (EMT)

**Fig. 2:**
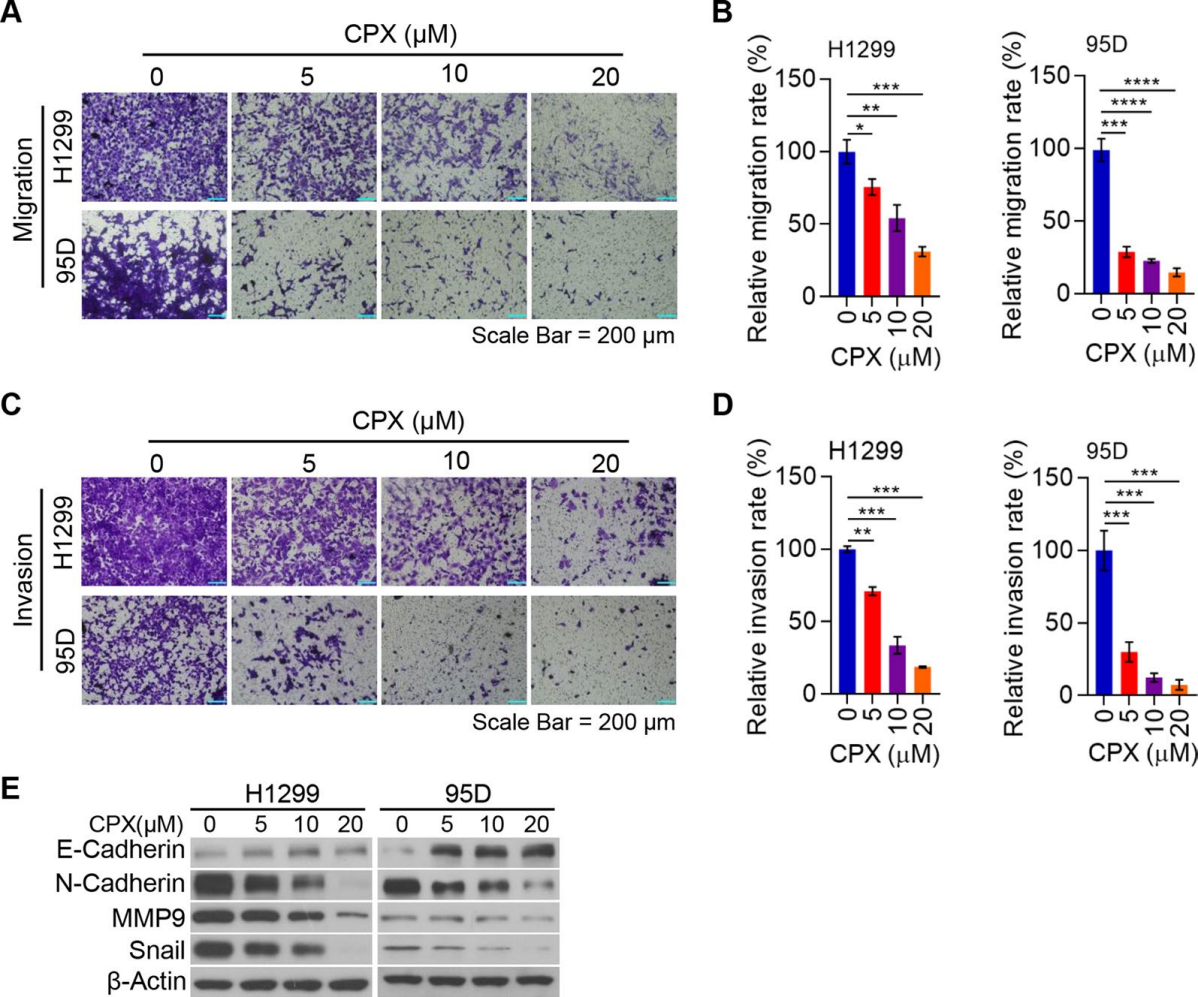
CPX suppresses NSCLC cell migration and invasion via inhibition of EMT. **A** and **B** H1299 and 95D cells with the treatment of CPX (0, 5, 10, and 20 μM) for 48 h. The migrated H1299 and 95D cells were stained with crystal violet solution and detected using a light microscope. Representative images of transwell migration assay were shown (Scale bar: 200 μm) (**A**). Cell migration rate quantified with ImageJ Plus (**B**). **C** and **D** H1299 and 95D cells treated with CPX (0, 5, 10, 20 μM) for 48 h. The invaded H1299 and 95D cells were stained with crystal violet solution and detected under a light microscope. Representative images of transwell invasion assay were shown (Scale bar: 200 μm) (**C**) and cell invasion rate quantified with ImageJ Plus (**D**). **E** H1299 and 95D cells with the treatment of CPX (0, 5, 10, 20 μM) for 48 h, cells were collected and the whole cell lysate was detected by Western blotting analysis. Data were presented as mean ± SD. (*for *p* < 0.05, **for *p* < 0.01, ***for *p* < 0.001, ****for *p* < 0.0001)

To elucidate the impact of CPX on NSCLC metastasis, a major risk factor for high mortality in NSCLC, we studied the effect of CPX on NSCLC cell migration. Our findings indicated that CPX significantly suppressed H1299 and 95D cell migration in a dose-dependent manner (Fig. 2A, B; Additional file 1: Fig. S3). Next, we examined the impact of CPX on the invasion ability of NSCLC cells. Our findings also showed that CPX prominently inhibited invasion ability of NSCLC cells in a dose-dependent manner (Fig. 2C, D). The EMT-related proteins in NSCLC cells with the treatment of CPX were examined to explore the underlying mechanism on how CPX suppresses the migration and invasion ability of NSCLC cells. We found that CPX treatment remarkably reduced the pre-metastatic and pro-invasive proteins such as MMP9, N-Cadherin and Snail in NSCLC cells (Fig. 2E). Consistently, the expression of anti-metastatic protein E-Cadherin increased (Fig. 2E). These findings indicated that CPX inhibits NSCLC cell migration and invasion through regulating EMT.

### CPX impairs mitochondrial functions and enhances the generation of ROS in NSCLC cells

**Fig. 3:**
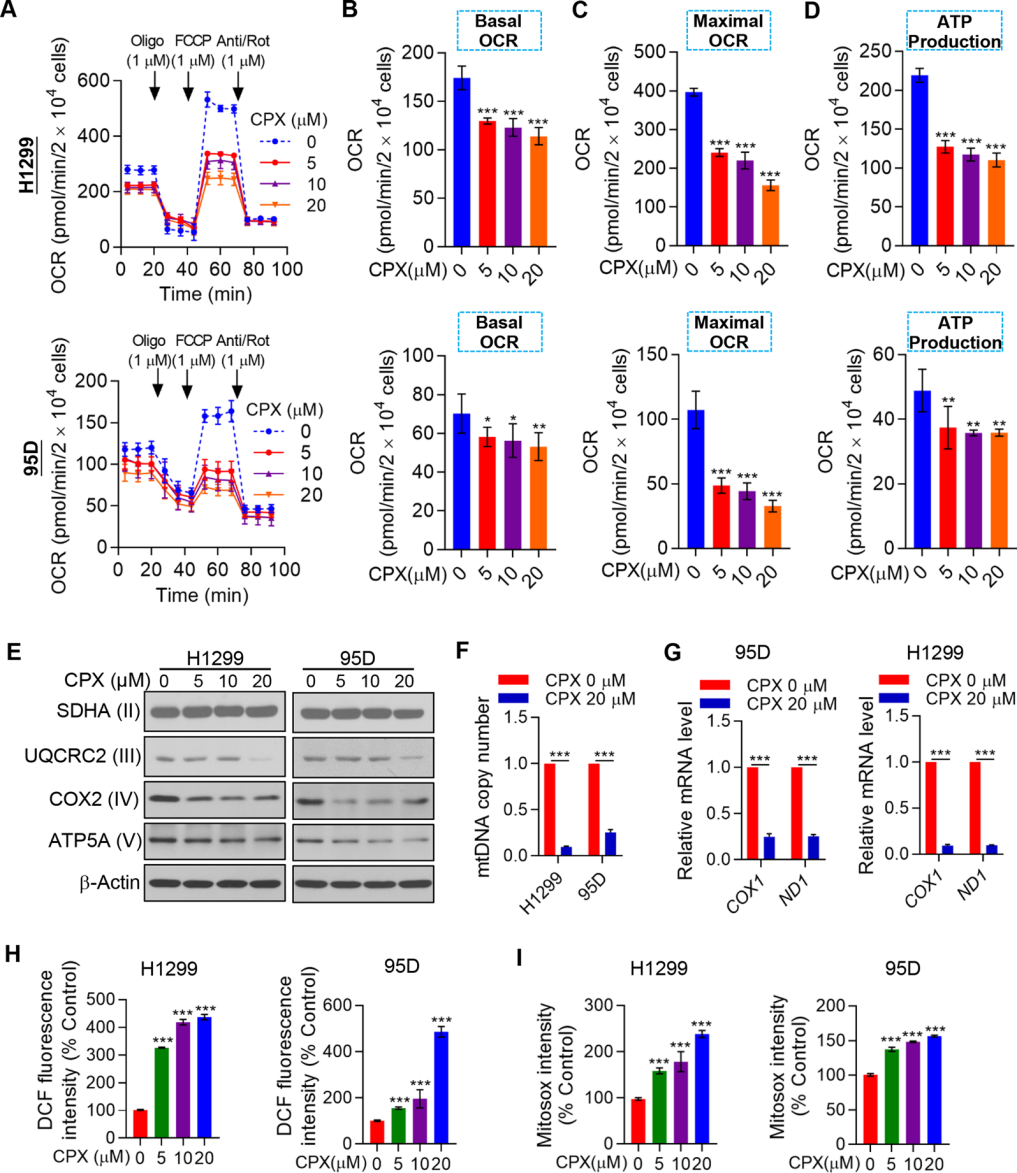
Mitochondrial respiration dysfunction and ROS induction in CPX-treated NSCLC cells. **A** The OCR in H1299 and 95D cells with the treatment of CPX (0, 5, 10, and 20 μM) assayed by Seahorse XF96 bioenergy analyzer. **B**–**D** The basal and maximal respiration (**B** and **C**), and the ATP production (**D**) were shown. **E** H1299 and 95D cells with the treatment of CPX (0, 5, 10, 20 μM) for 48 h, and cells were collected and the whole cell lysate was detected by Western blotting analysis. **F** H1299 and 95D cells with the treatment of CPX (0, 5, 10 and 20 μM) and mitochondrial DNA number measured. **G** The mRNA expression levels of *COX1* and *ND1* in H1299 and 95D cells with the treatment of vehicle control (DMSO) or CPX (20 μM). **H** H1299 and 95D cells with the treatment of CPX (0, 5, 10, 20 μM) and total cellular reactive oxygen species level measured. **I** H1299 and 95D cells with the treatment of CPX (0, 5, 10, 20 μM) and mitochondrial reactive oxygen species (MitoSOX) level measured. Data were presented as mean ± SD (n = 3, *for *p* < 0.05, **for *p* < 0.01, ***for *p* < 0.001)

Mitochondria produce the majority of cellular energy in the form of ATP needed for cell survival. Therefore, targeting mitochondrial oxidative phosphorylation (OXPHOS) has been thought to be a promising cancer treatment strategy. We evaluated OCR changes in H1299 and 95D cells treated with CPX (Fig. 3A). To understand the detailed indicators of mitochondrial respiration, OCR data of different time points were analyzed. Combined with analysis of other parameters of mitochondrial function, it suggested that CPX significantly reduced basal and maximal respiration, as well as the ATP production in NSCLC cells (Fig. 3B–D). Decreased protein levels of mitochondrial respiratory chain enzyme subunit UQCRC2, COX 2, and ATP5A in the NSCLC cells treated with CPX were also found (Fig. 3E). Furthermore, CPX may cause a decrease in mtDNA copy numbers in H1299 and 95D cells (Fig. 3F), and a decrease of the mRNA levels of *COX 1* and *ND1,* which are encoded by mtDNA (Fig. 3G).

In the eukaryotic cells, mitochondria produce most of ROS. From the results of the OCR of the NSCLC cells markedly inhibited by CPX (Fig. 3A–D), we could find that mitochondrial dysfunction happened when the cells were treated with CPX. CPX treatment markedly decreased the protein expression of complex III subunit: UQCRC2; complex IV subunit: COX 2; and complex V subunit: ATP5A; as well as the decrease in mtDNA copy number and mRNA levels of *COX1* (complex IV) and *ND1* (complex I) (Fig. 3E–G), we speculated that mitochondrial dysfunction induced by CPX might enhance the production of ROS. We found that CPX remarkably increased the production of intracellular and mitochondrial ROS in NSCLC cells (Fig. 3H, I).

Collectively, these results suggest that mitochondrial respiration interruption and increase of ROS might contribute to CPX-induced growth inhibition of the NSCLC cells.

### CPX significantly promotes NSCLC cell aerobic glycolysis

**Fig. 4:**
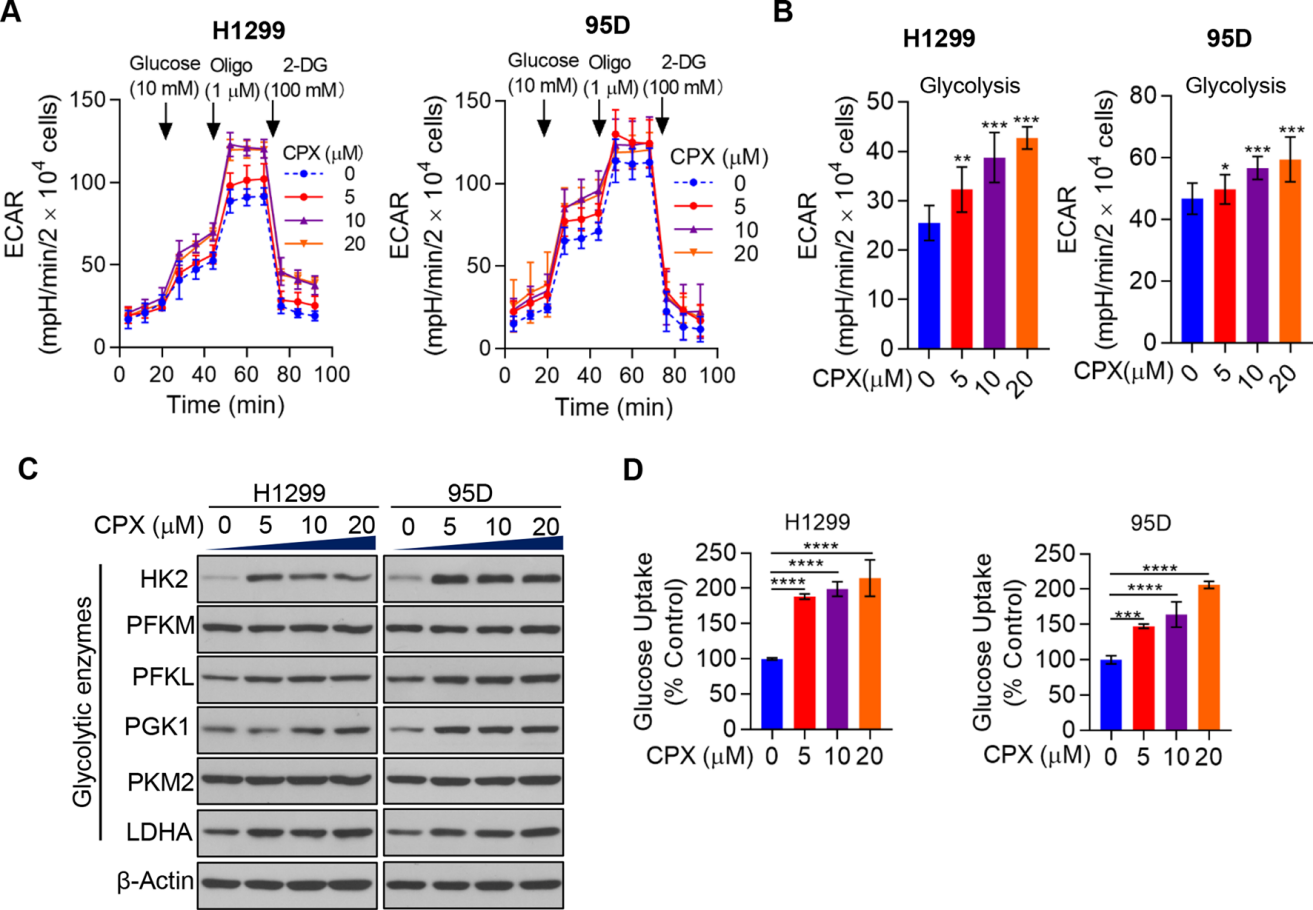
CPX treatment promotes aerobic glycolysis in NSCLC cells. **A** Alteration of ECAR in H1299 and 95D cells with the treatment of CPX (0, 5, 10, 20 μM). **B** Glycolytic capacity of NSCLC cells with the treatment of CPX (0, 5, 10, 20 μM). **C** H1299 and 95D cells with the treatment of CPX (0, 5, 10, 20 μM), and cells were collected and the whole cell lysate was examined by Western blot analysis. **D** Glucose uptake in H1299 and 95D cells treated with CPX (0, 5, 10, 20 μM)**.** Data were presented as mean ± SD (n = 3, **for *p* < 0.01, ***for *p* < 0.001, ****for *p* < 0.0001)

We next explored the effect of CPX on aerobic glycolysis in NSCLC cells. Strikingly, the results showed that CPX markedly increased the aerobic glycolysis rate of the NSCLC cell lines H1299 and 95D (Fig. 4A, B). To clarify in depth the effect of CPX on aerobic glycolysis of NSCLC cells, the expressions level of some key glycolytic enzymes HK2, PFKL, PGK1 and LDHA were detected. It showed that the protein levels increased significantly when treated with CPX compared with that of vector-treated NSCLC cells (Fig. 4C). We determined the glucose uptake level in DMSO and CPX treated NSCLC cells to confirm the role of CPX in aerobic glycolysis. It turned out that CPX treatment remarkably promoted glucose uptake compared to that in DMSO treated NSCLC cells (Fig. 4D**)**. These findings indicated that CPX played an important role in promotion of aerobic glycolysis in NSCLC cells, at least partially through increasing the expression of glycolytic enzymes.

### CPX induces PERK-dependent ER stress to activate apoptosis in NSCLC cells

**Fig. 5:**
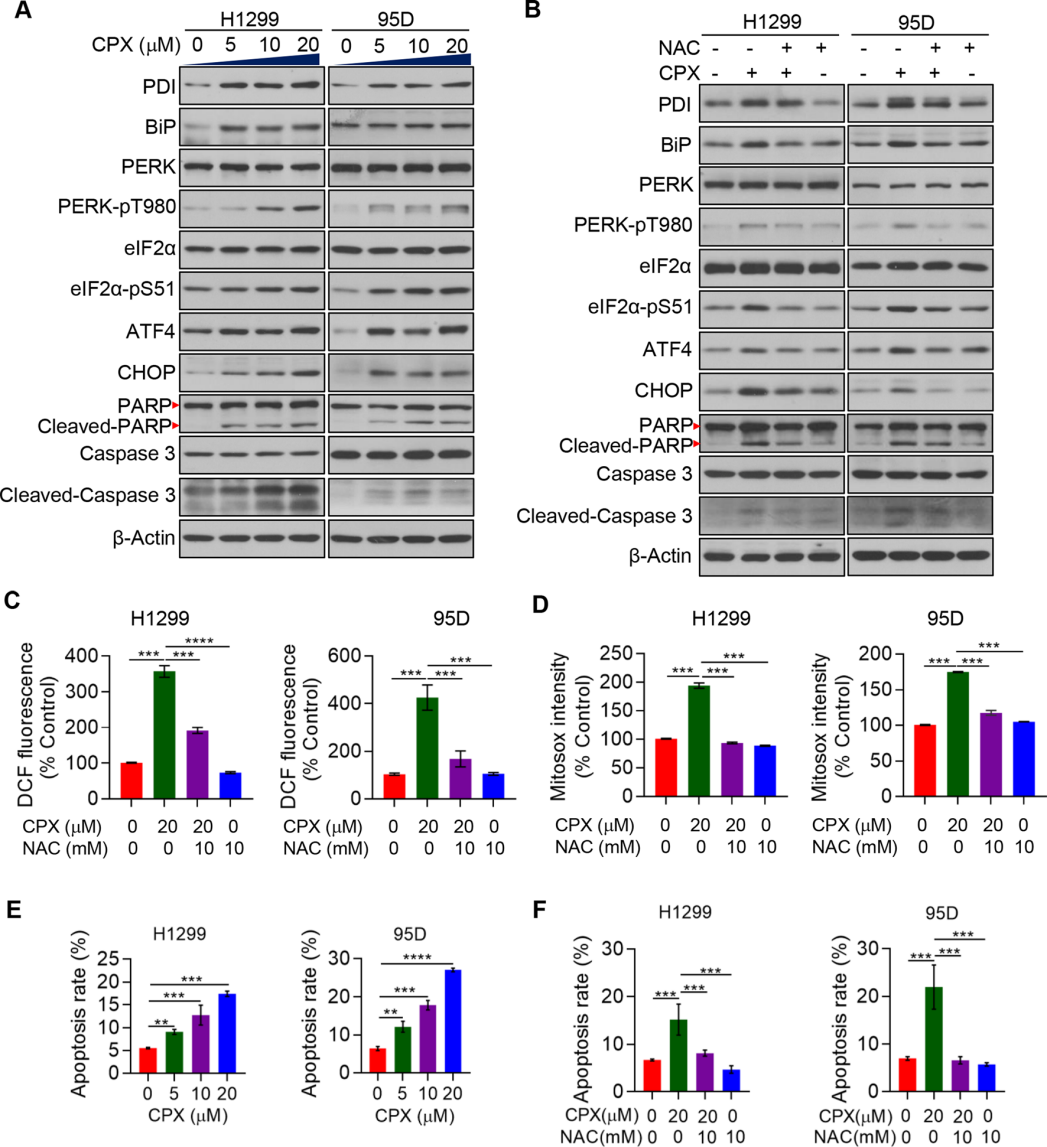
PERK-dependent ER stress activation and apoptosis induction by CPX. **A** The expression of ER stress and apoptosis related proteins in H1299 and 95D cells with the treatment of CPX (0, 5, 10 and 20 μM) and detected by Western blotting. **B** H1299 and 95D cells with the treatment of CPX (0 and 20 μM), CPX (20 μM) combined with 10 mM NAC or 10 mM NAC alone for 48 h. Western blotting was used to detect proteins related to ER stress and apoptosis. **C** H1299 and 95D cells with the treatment of CPX (0 and 20 μM), CPX (20 μM) combined with 10 mM NAC or 10 mM NAC alone for 48 h, and the Intracellular ROS level was determined. **D** H1299 and 95D cells with the treatment of CPX (0 and 20 μM), CPX (20 μM) combined with 10 mM NAC or 10 mM NAC alone for 48 h, and mitochondrial ROS was determined. **E** H1299 and 95D cells with the treatment of CPX (0, 5, 10, 20 μM) for 48 h, apoptosis rate was detected. **F** H1299 and 95D cells with the treatment of CPX (0 and 20 μM), CPX (20 μM) combined with the 10 mM NAC or 10 mM NAC alone for 48 h, apoptosis rate was detected. Data were presented as mean ± SD (n = 3, **for *p* < 0.01, ***for *p* < 0.001, ****for *p* < 0.0001)

We further investigated the impact of CPX on ER stress and UPR^ER^ in NSCLC cells to understand the molecular mechanism by which CPX suppresses NSCLC cell growth. Our results revealed that CPX activated PERK-dependent ER stress and UPR^ER^ in NSCLC cells, and the CPX treatment dramatically increases the phosphorylation at Threonine 980 of PERK and Serine 51 of eIF2α, respectively, both in dose-dependent manners (Fig. 5A). Our results further revealed increased expressions of ATF4, BiP, CHOP, and PDI proteins when treated with CPX (Fig. 5A). In addition, pretreatment of the NSCLC cells with NAC, a ROS scavenging agent, would rescue the CPX’s effects on the expressions of ATF4, PDI, BiP, CHOP and PDI, as well as the activation of both PERK and eIF2α (Fig. 5B). These results showed that the treatment with CPX would activate PERK-dependent ER stress and UPR^ER^ in NSCLC cells that are in ROS-dependent manners. NAC pretreatment could remarkably attenuate the effect of CPX on promotion of both cellular and mitochondrial ROS production in the NSCLC cells (Fig. 5C, D). It could be presumed that CPX may induce apoptosis in NSCLC cells through the activation of ER stress and UPR^ER^. Herein, we demonstrated that CPX treatment led to the cleavage of both PARP and Caspase-3 (Fig. 5A) and induction of apoptosis (Fig. 5E) in NSCLC cells in a dose-dependent manner, while pretreatment of the NSCLC cells with NAC rescued the CPX-induced apoptosis (Fig. 5F). The addition of Z-VAD-FMK, a cell-permeable pan-caspase inhibitor, blocked the cleavage of both PARP and Caspase 3, which further confirmed that CPX induced apoptosis in NSCLC cells (Additional file 1: Fig. S4).Together, these findings reveal that CPX inhibits the growth of NSCLC cells, at least in part, by way of activation of PERK-dependent ER stress and UPR^ER^ to induce apoptosis.

### CPX dramatically suppresses *in vivo* NSCLC cell growth

**Fig. 6:**
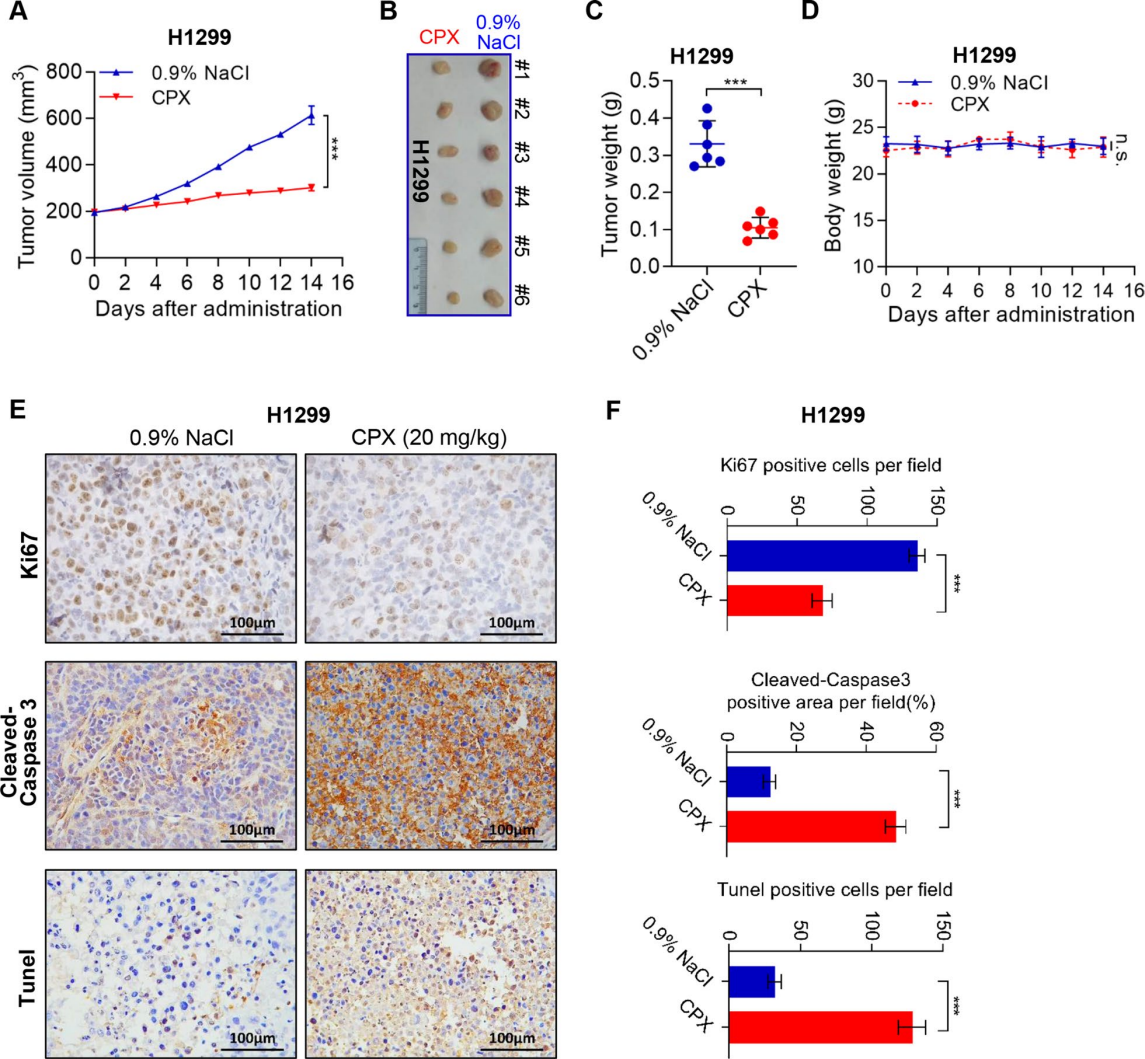
Tumor growth inhibition by CPX in an NSCLC mouse xenograft model. **A**–**D** Tumor-bearing nude mice (6 mice per group) were injected intraperitoneally with either CPX (20 mg/kg) or 0.9% NaCl, respectively. Data were presented as mean ± SD (n = 6, ***for *p* < 0.001, ns: no significance for indicated comparison). Tumor volumes evaluated (**A**) after 14 days of continuous injection, the images of dissected tumors from tumor-bearing mice were shown (**B**) and the tumor weight was measured (**C**). Changes in the mean body weight in the two groups of CPX or 0.9% NaCl injection xenograft model (**D**). **E** and **F** Representative images of IHC staining of Ki67, Cleaved-Caspase 3, and Tunel in the tumor of NSCLC mouse xenograft model injected intraperitoneally with 0.9% NaCl or CPX (20 mg/kg) (**E**), and Ki67, Cleaved-Caspase 3 and TUNEL-positive staining cells in the tumor sections were quantified by ImageJ Plus (**F**). Data were presented as mean ± SD (n = 6; ***for *p* < 0.001; ns = not significant)

To validate the *in vitro* results, we then use a mouse xenograft model to investigate possible tumor suppressive characteristics and antitumor activity of CPX *in vivo*. Similar to the *in vitro* results, compared with the control group, CPX remarkably inhibited NSCLC cell growth *in vivo* (Fig. 6A, B). Moreover, CPX also significantly decreased the tumor weight compared to control (0.9% NaCl) (Fig. 6C). Notably, the mice were well tolerated to CPX and without notable body weight loss was observed throughout the course of CPX treatment (Fig. 6D). IHC staining shows that CPX treatment reduced proliferation (Ki67 staining) and increases apoptosis (Cleaved-Caspase 3 and Tunel staining) in NSCLC xenograft tumor. These results indicate that CPX treatment significantly suppressed NSCLC cell growth *in vivo* (Fig. 6E, F).

## Discussion

Our current study has explored the anti-tumor activity of CPX in NSCLC, we found that CPX remarkably inhibited proliferation and colony formation ability of H1299 and 95D cells in a dose-dependent manner. Mechanistically, CPX treatment reduced the CDK4 and Cyclin D1 protein levels, thus inducing cell cycle arrest at G1 phase, inhibiting Rb phosphorylation, and increasing p21 expression in NSCLC to suppress cell proliferation. These findings indicated that CPX could inhibit NSCLC cells growth *in vitro*. Furthermore, an *in vivo* experiment with a xenograft tumor model demonstrated that CPX treatment could dramatically inhibit tumor growth of NSCLC. In addition, CPX treatment remarkably reduced Ki67 (a cell proliferation marker) expression in tumor tissues of NSCLC xenograft mouse models, compared with that of 0.9% NaCl treatment.

As an initial stage of metastasis, EMT is an essential process of cancer cell migration, invasion and apoptosis resistance [23–25]. The mechanism of snail-induced EMT includes E-Cadherin inhibition and N-Cadherin activation, thereby reducing cell adhesion and promoting migration [26, 27]. In this study, CPX treatment induced E-Cadherin up-regulation; however, the expression levels of N-Cadherin, MMP9 and Snail, were remarkably downregulated. These data indicated the abilities of migration and invasion in NSCLC cells were suppressed through inhibition of EMT [28]. Consistently, in this study, the notable cell invasion and migration inhibition abilities of CPX were confirmed by the transwell assay.

Cancer cells frequently exhibit metabolic abnormalities in hyperglycolysis (known as the Warburg effect or aerobic glycolysis), even at normal oxygen concentrations, which is widely recognized as one of the hallmarks of cancer and a promising drug target for anticancer therapy [29]. In the present study, our findings demonstrated that CPX significantly suppressed OXPHOS in NSCLC cells. However, aerobic glycolysis in H1299 and 95D cells is increased. Therefore, targeted cell bioenergetics would generate effective and feasible therapeutic strategies for the NSCLC treatment. In addition, we found that the protein level of COX2, one of the three mtDNA encoded subunits of cytochrome c oxidase, was notably decreased in response to CPX treatment in NSCLC cells. Moreover, mtDNA content was also significantly reduced and the mRNA levels of two mtDNA encoded genes *COX 1* and *ND1* were both decreased. Our results indicate that CPX could suppress the replication of mtDNA and gene transcription, and thus impairing the integrity of respiratory enzyme complexes in the mitochondrial respiratory chain, and resulting in OXPHOS dysfunction. Furthermore, our results showed the nuclear DNA encoded proteins UQCRC2 and ATP5A were also reduced, which suggest that the defects in mtDNA replication and gene transcription activate the retrograde signaling from mitochondria to nucleus, therefore alleviating the substrate burden on the overwhelmed protein homeostasis in stressed mitochondria. In addition, in line with increased aerobic glycolysis in NSCLC cells, increased expression of several key glycolytic enzymes was observed in NSCLC cells treated with CPX. It was also discovered that CPX treatment led to glucose uptake dramatically increased, suggesting that CPX may promote glucose uptake to increase aerobic glycolysis.

Induction of apoptosis by activating ER Stress and UPR^ER^ is considered an effective therapy for cancers. ER plays an essential role in the surveillance of protein quality control ensuring the correct protein folding and degradation of abnormal proteins, this process is termed ER-associated degradation (ERAD) [30]. The PERK-eIF2α signaling pathway is essential in regulating ER stress mediated apoptosis after virus infection as well as sensitizing TRAIL-induced cell death in colon cancer cells [31–33]. We previously demonstrated that CPX activates PERK-mediated ER stress to activate apoptosis in CRC [7]. Since the accumulation of misfolded proteins in the ER lumen leads to ER stress which subsequently results in PERK autophosphorylation through a conformational change and dimerization. Following after autophosphorylation, PERK becomes an active kinase with the ability to phosphorylate eIF2α. Once phosphorylated, eIF2 α can then activate a downstream factor, ATF4, which stimulates CHOP expression afterwards [34]. In this study, we further demonstrated the effect of CPX on ER stress and UPR^ER^ signaling pathway in NSCLC cells. Furthermore, we found that CPX impaired mitochondrial respiration via reducing subunits of the mitochondrial complex, which in turn led to increasing both mitochondrial and cellular ROS levels, therefore promoting cell death in NSCLC cells. Although ER stress is essential for cell survival, high levels of or prolonged ER stress can trigger apoptosis [15, 19]. However, we do not know whether CPX induced ER stress also have contributed directly to the induction of apoptosis as ROS in NSCLC cells. Our finding further showed that NAC (a ROS scavenger) could rescue the cell apoptosis induced by CPX, indicates that ROS may contribute to the anticancer activity of CPX. Collectively, our findings demonstrated a critical role for ROS and PERK-eIF2α-ATF4-CHOP signaling pathway in CPX induced apoptosis in NSCLC cells. Further studies will be needed to validate our hypothesis and understand the molecular basis for CPX activated ER stress in triggering apoptosis of NSCLC cells.

## Conclusions

**Fig. 7:**
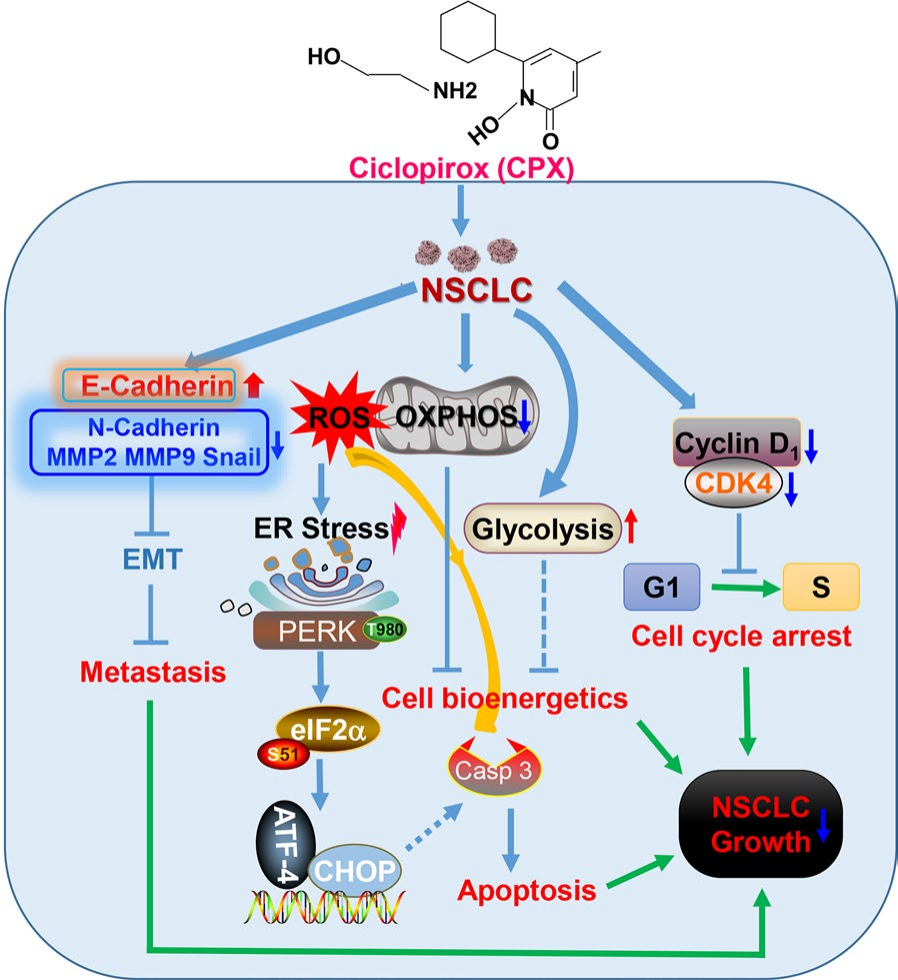
Mechanisms of NSCLC cell proliferation, migration and invasion inhibition by CPX. CPX shows anti-tumor activities in NSCLC through arresting cell cycle, suppressing cell migration and invasion via the inhibition of EMT. CPX induces ROS generation, impairs mitochondrial respiration, and increases glycolysis capacity. The anticancer activity of CPX also depends on ROS and/or ROS-induced ER stress through PERK-eIF2α-ATF4-CHOP signaling pathway to initiate apoptosis in NSCLC cells

We present here a mechanistic model of how CPX inhibits the proliferation, migration, and invasion in NSCLC cells, as well as inducing and activating ER stress and UPR^ER^ signaling pathway which may induce apoptosis in NSCLC cells and inhibiting NSCLC progression (Fig. 7). In summary, our findings identify a novel molecular mechanism by which CPX suppresses NSCLC cell growth both *in vitro* and *in vivo*, and support the re-purposing potential of CPX for treatment of NSCLC patients.

## Supplementary Information

**Additional file 1.** Supplementary Figures (Figure S1–S4) and supplementary Tables (Table S1–S3).

## Data Availability

Not applicable.

## Abbreviations

ATF4: Activating transcription factor 4 (ATF4)
CCK-8: Cell Counting Kit-8
CHOP: C/EBP homologous protein
CPX: Ciclopirox olamine
DCFH-DA: 2’,7’-Dichlorodihydrofluorescein diacetate
ECAR: Extracellular acidification rate
eIF2α: The alpha-subunit of eukaryotic initiation factor 2
EMT: Epithelial-to-mesenchymal transition
ER: Endoplasmic reticulum
mtDNA: Mitochondrial DNA
NAC: N-acetyl-L-cysteine
NSCLC: Non‐small‐cell lung cancer
OCR: Oxygen consumption rate
OXPHOS: Oxidative phosphorylation
PERK: Protein kinase RNA-like endoplasmic reticulum kinase
PI: Propidium iodide
qPCR: Quantitative real-time polymerase chain reaction
ROS: Reactive oxygen species
SCLC: Small‐cell lung cancer
UPR: Unfolded protein response

## References

[1] Sung H, Ferlay J, Siegel RL, Laversanne M, Soerjomataram I, Jemal A, Bray F (2021). Global cancer statistics 2020: GLOBOCAN estimates of incidence and mortality worldwide for 36 cancers in 185 countries. CA Cancer J Clin.

[2] Bareschino MA, Schettino C, Rossi A, Maione P, Sacco PC, Zeppa R, Gridelli C (2011). Treatment of advanced non small cell lung cancer. J Thorac Dis.

[3] Sher T, Dy GK, Adjei AA (2008). Small cell lung cancer. Mayo Clin Proc.

[4] Wu Q, Zhang B, Li B, Cao X, Chen X, Xue Q (2020). PTBP3 promotes migration of non-small cell lung cancer through regulating E-cadherin in EMT signaling pathway. Cancer Cell Int.

[5] Herbst RS, Morgensztern D, Boshoff C (2018). The biology and management of non-small cell lung cancer. Nature.

[6] Eberhard Y, McDermott SP, Wang X, Gronda M, Venugopal A, Wood TE, Hurren R, Datti A, Batey RA, Wrana J (2009). Chelation of intracellular iron with the antifungal agent ciclopirox olamine induces cell death in leukemia and myeloma cells. Blood.

[7] Qi J, Zhou N, Li L, Mo S, Zhou Y, Deng Y, Chen T, Shan C, Chen Q, Lu B (2020). Ciclopirox activates PERK-dependent endoplasmic reticulum stress to drive cell death in colorectal cancer. Cell Death Dis.

[8] Shen T, Huang S (2016). Repositioning the old fungicide ciclopirox for new medical uses. Curr Pharm Des.

[9] Yang J, Milasta S, Hu D, AlTahan AM, Interiano RB, Zhou J, Davidson J, Low J, Lin W, Bao J (2017). Targeting histone demethylases in MYC-driven neuroblastomas with ciclopirox. Cancer Res.

[10] Weir SJ, Patton L, Castle K, Rajewski L, Kasper J, Schimmer AD (2011). The repositioning of the anti-fungal agent ciclopirox olamine as a novel therapeutic agent for the treatment of haematologic malignancy. J Clin Pharm Ther.

[11] Sen S, Hassane DC, Corbett C, Becker MW, Jordan CT, Guzman ML (2013). Novel mTOR inhibitory activity of ciclopirox enhances parthenolide antileukemia activity. Exp Hematol.

[12] Su Z, Han S, Jin Q, Zhou N, Lu J, Shangguan F, Yu S, Liu Y, Wang L, Lu J (2021). Ciclopirox and bortezomib synergistically inhibits glioblastoma multiforme growth via simultaneously enhancing JNK/p38 MAPK and NF-κB signaling. Cell Death Dis.

[13] Lu T, Tang J, Shrestha B, Heath BR, Hong L, Lei YL, Ljungman M, Neamati N (2020). Up-regulation of hypoxia-inducible factor antisense as a novel approach to treat ovarian cancer. Theranostics.

[14] Huang Z, Huang S (2021). Reposition of the fungicide ciclopirox for cancer treatment. Recent Pat Anticancer Drug Discov.

[15] Hetz C, Papa FR (2018). The unfolded protein response and cell fate control. Mol Cell.

[16] Maly DJ, Papa FR (2014). Druggable sensors of the unfolded protein response. Nat Chem Biol.

[17] Jaronen M, Goldsteins G, Koistinaho J (2014). ER stress and unfolded protein response in amyotrophic lateral sclerosis-a controversial role of protein disulphide isomerase. Front Cell Neurosci.

[18] Walter P, Ron D (2011). The unfolded protein response: from stress pathway to homeostatic regulation. Science.

[19] Tabas I, Ron D (2011). Integrating the mechanisms of apoptosis induced by endoplasmic reticulum stress. Nat Cell Biol.

[20] Li X, Zheng J, Chen S, Meng FD, Ning J, Sun SL (2021). Oleandrin, a cardiac glycoside, induces immunogenic cell death via the PERK/elF2α/ATF4/CHOP pathway in breast cancer. Cell Death Dis.

[21] Li Y, Lu J, Chen Q, Han S, Shao H, Chen P, Jin Q, Yang M, Shangguan F, Fei M (2019). Artemisinin suppresses hepatocellular carcinoma cell growth, migration and invasion by targeting cellular bioenergetics and Hippo-YAP signaling. Arch Toxicol.

[22] Lan L, Wei W, Zheng Y, Niu L, Chen X, Huang D, Gao Y, Mo S, Lu J, Guo M (2018). Deferoxamine suppresses esophageal squamous cell carcinoma cell growth via ERK1/2 mediated mitochondrial dysfunction. Cancer Lett.

[23] Kalluri R, Weinberg RA (2009). The basics of epithelial-mesenchymal transition. J Clin Investig.

[24] Thiery JP, Acloque H, Huang RY, Nieto MA (2009). Epithelial-mesenchymal transitions in development and disease. Cell.

[25] Nieto MA, Huang RY, Jackson RA, Thiery JP (2016). EMT: 2016. Cell.

[26] Lamouille S, Xu J, Derynck R (2014). Molecular mechanisms of epithelial-mesenchymal transition. Nat Rev Mol Cell Biol.

[27] Puisieux A, Brabletz T, Caramel J (2014). Oncogenic roles of EMT-inducing transcription factors. Nat Cell Biol.

[28] Zhang Y, Zhang X, Ye M, Jing P, Xiong J, Han Z, Kong J, Li M, Lai X, Chang N (2018). FBW7 loss promotes epithelial-to-mesenchymal transition in non-small cell lung cancer through the stabilization of Snail protein. Cancer Lett.

[29] Koppenol WH, Bounds PL, Dang CV (2011). Otto Warburg's contributions to current concepts of cancer metabolism. Nat Rev Cancer.

[30] Lemberg MK, Strisovsky K (2021). Maintenance of organellar protein homeostasis b yER-associated degradation and related mechanisms. Mol Cell.

[31] Baer A, Lundberg L, Swales D, Waybright N, Pinkham C, Dinman JD, Jacobs JL, Kehn-Hall K (2016). Venezuelan equine encephalitis virus induces apoptosis through the unfolded protein response activation of EGR1. J Virol.

[32] Zhao Y, Hu N, Jiang Q, Zhu L, Zhang M, Jiang J, Xiong M, Yang M, Yang J, Shen L (2021). Protective effects of sodium butyrate on rotavirus inducing endoplasmic reticulum stress-mediated apoptosis via PERK-eIF2α signaling pathway in IPEC-J2 cells. J Anim Sci Biotechnol.

[33] Lee SJ, Lee DE, Choi SY, Kwon OS (2021). OSMI-1 enhances TRAIL-induced apoptosis through ER stress and NF-κB signaling in colon cancer cells. Int J Mol Sci.

[34] Gonzalez-Teuber V, Albert-Gasco H, Auyeung VC, Papa FR, Mallucci GR, Hetz C (2019). Small molecules to improve ER proteostasis in disease. Trends Pharmacol Sci.

