# Supplementary material for: Ciclopirox targets cellular bioenergetics and activates ER stress to induce apoptosis in non-small cell lung cancer cells
Source: Cell Commun Signal. 2022 Mar 24;20:37. doi: 10.1186/s12964-022-00847-x (PMC8943949; doi:10.1186/s12964-022-00847-x)
Supplement: Supplementary file 2 — Additional file 1. Supplementary Figures (Figure S1–S4) and supplementary Tables (Table S1–S3). [file 12964_2022_847_MOESM2_ESM.pdf]

Supplementary information

Ciclopirox Targets Cellular Bioenergetics and Activates ER Stress to Induce Apoptosis in Non-small Cell Lung Cancer Cells

Junwan Lu, Yujie Li, Shiwei Gong, Jiaxin Wang, Xiaoang Lu, Qiumei Jin, Bin Lu, Qin Chen

Figures

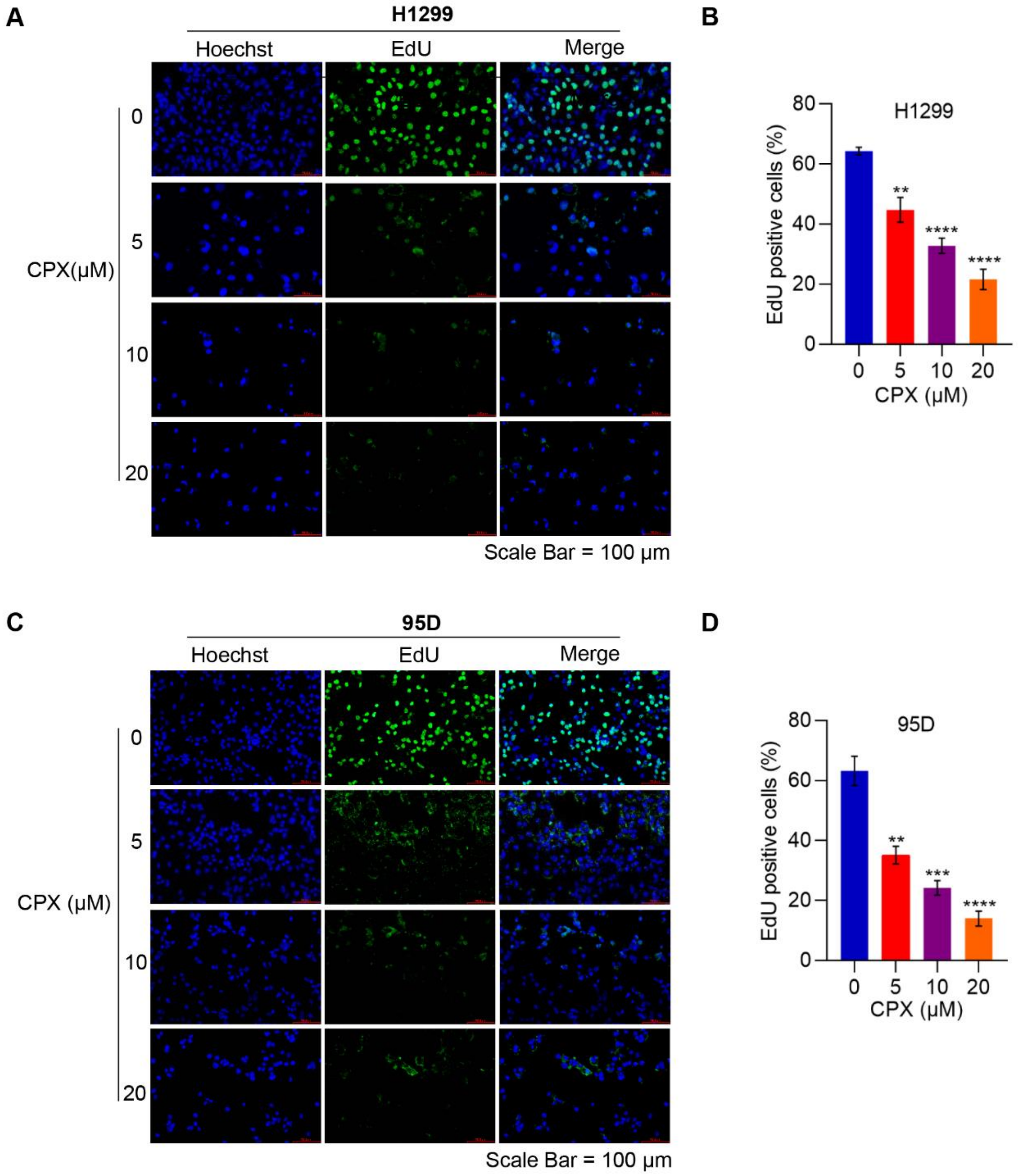

Figure S1 CPX inhibited human NSCLC cell proliferation *in vitro*. Fluorescence micrographs of CPX (0, 5, 10, and 20 μM, 24 h)-treated H1299 and 95D cells with EdU incorporation. Green, EdU-positive cells; Blue, Hoechst 33342 for nuclear staining. Scale bar, 100 μm. (**for $p < 0.01$,***for $p < 0.001$, ****for $p < 0.0001$).

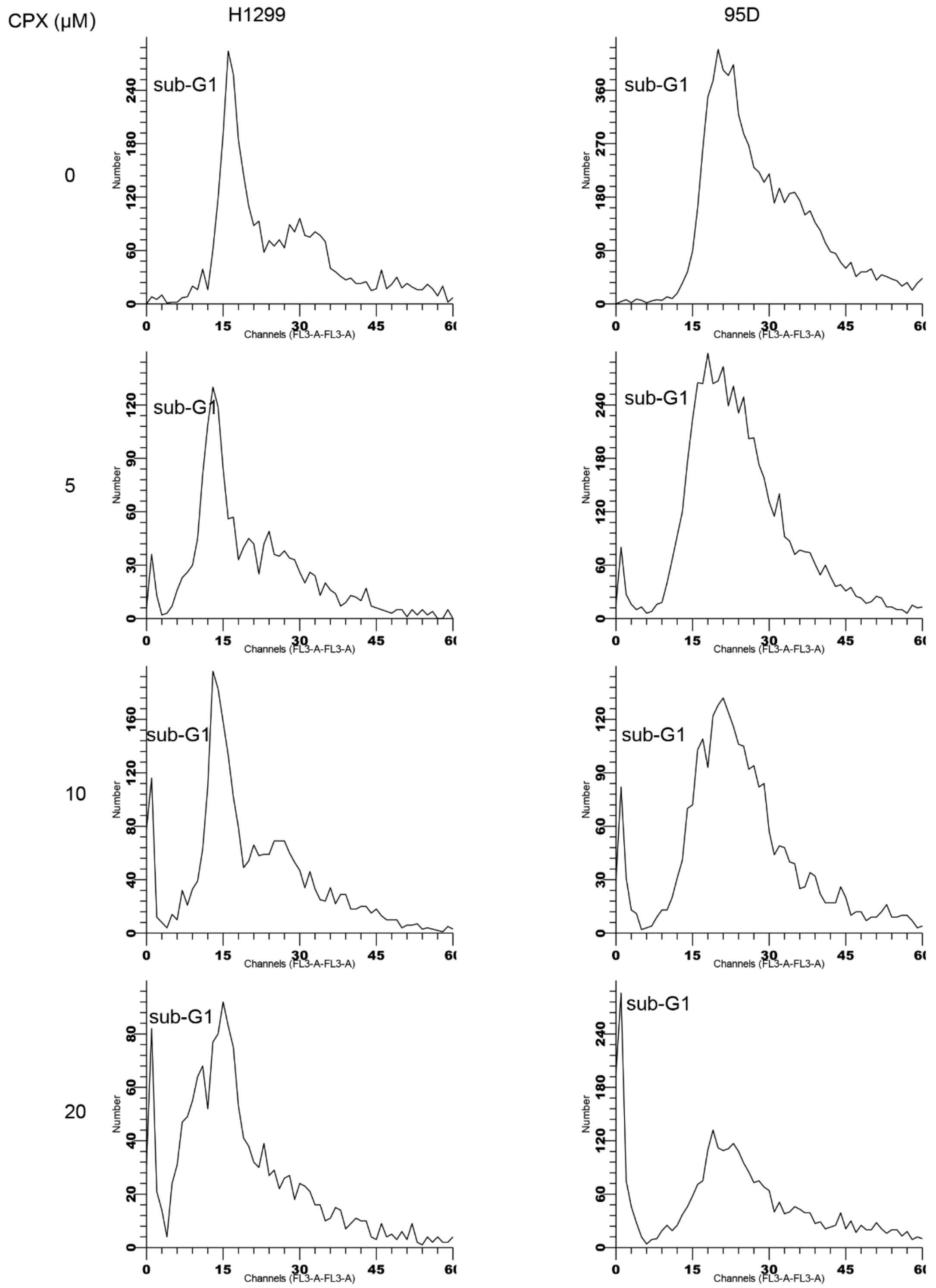

Figure S2 Cell cycle distribution assay. H1299 or 95D cells were incubated in the medium containing DMSO (vehicle control) or CPX (5, 10, 20 μM) for 48 h. Cell cycle distribution was analyzed by flow cytometry. All cells were included in the analysis.

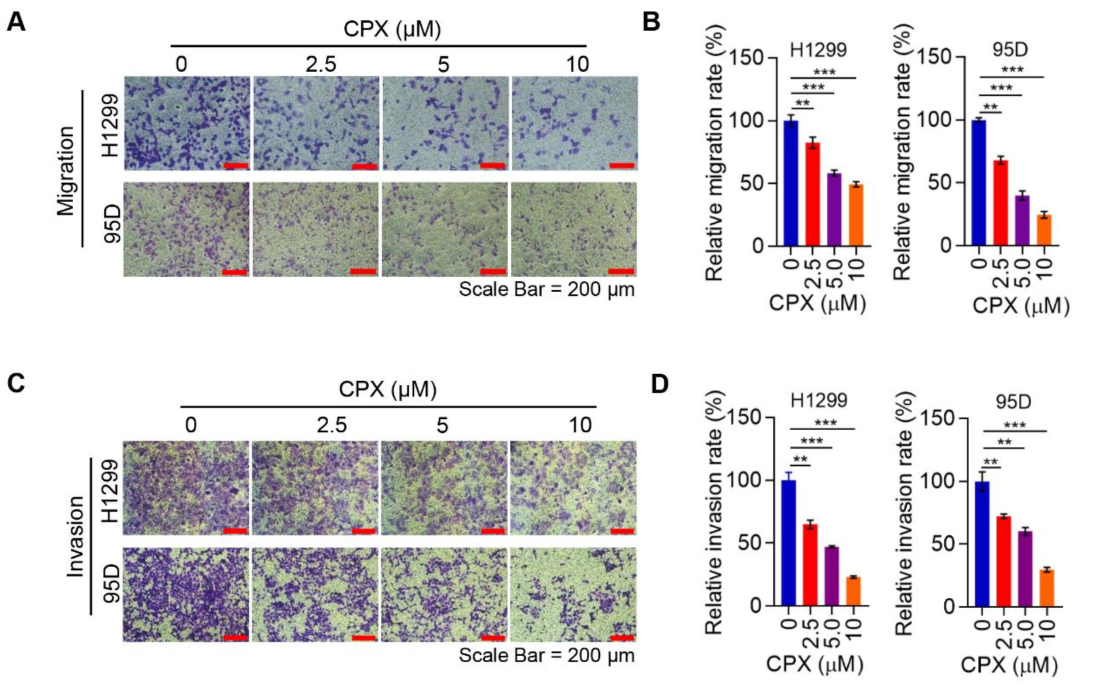

Figure S3 CPX suppresses NSCLC cell migration and invasion. A and **B** H1299 and 95D cells with the treatment of CPX (0, 2.5, 5, and 10 μM) for 48 h. The migrated H1299 and 95D cells were stained with crystal violet solution and detected using a light microscope. Representative images of transwell migration assay were shown (Scale bar: 200 μm) (**A**). Cell migration rate quantified with ImageJ Plus (**B**). **C** and **D** H1299 and 95D cells treated with CPX (0, 2.5, 5, and 10 μM) for 48 h. The invaded H1299 and 95D cells were stained with crystal violet solution and detected under a light microscope. Representative images of transwell invasion assay were shown (Scale bar: 200 μm) (**C**) and cell invasion rate quantified with ImageJ Plus (**D**). (**for $p < 0.01$, ***for $p < 0.001$).

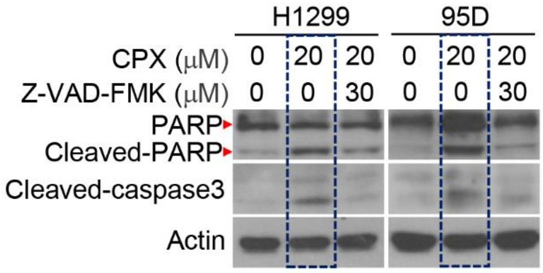

Figure S4 CPX induced apoptosis in NSCLC through Caspase3-PARP pathway. H1299 and 95D cells treated with CPX (0 and 20 μM), CPX (20 μM) combined with 30 mM Z-VAD-FMK for 48 h. Western blotting was used to detect the proteins as indicated.

Tables

Table S1 Sequences of primers used in qPCR analysis for selected genes.

**Genes**	**Forward 5'-3'**	**Reverse 5'-3'**
ND1	TTCCTAATGCTTACCGAACGA	AGAAGAGCGATGGTGAGAGC
COX I	CAGAACGCAACCTCAACAC	TCCGAATGGTAGGATAA
β-ACTIN	AGCACAGAGCCTCGCCTTTG	AAGCCGGCCTTGCACATG

Table S2 Sequences of primers used in qPCR for mtDNA copy number analysis.

**Genes**	**Forward 5’-3’**	**Reverse 5’-3’**
CYT B	CCC CAC AAA CCC CAT TAC TAA ACC CA	TTT CAT CAT GCG GAG ATG TTG GAT GG
18S rRNA	TAG AGG GAC AAG TGG CGT TC	CGC TGA GCC AGT CAG TGT

Table S3 Details of the antibodies used for Western-blot and IHC analyses.

Antibodies	SOURCE	Cat #
Anti-ATF4	Abcam	ab184909
Anti-COXII	Abcam	ab110258
Anti-Ki67	Abcam	ab181064
Anti-PFKL	Abcam	ab92742
Anti-PKM2	Abcam	ab154804
Anti-Cyclin D1	Cell signaling Technology	55506
Anti-Caspase 3	Cell signaling Technology	9662
Anti-Cleved-Caspase 3	Cell signaling Technology	9664
Anti-Bip	Cell signaling Technology	3177
Anti-CHOP	Cell signaling Technology	5554
Anti-p-Rb	Cell signaling Technology	8516
Anti-N-Cadherin	Cell signaling Technology	13116
Anti-PDI	Cell signaling Technology	3501
Anti-PERK	Cell signaling Technology	5683
Anti-PERK-pT980	Cell signaling Technology	12185
Anti-eIF2α	Cell signaling Technology	5324
Anti-eIF2α-pS51	Cell signaling Technology	3597
Anti-PARP	Cell signaling Technology	9532
Anti-Snail	Cell signaling Technology	3879
Anti-p21	Cell signaling Technology	2947
Anti-LDHA	Zen Bioscience	501146
Anti-HK2	Zen Bioscience	220458
Anti-PFKM	Zen Bioscience	505477
Anti-MMP2	Santa Cruz Biotechnology	13595
Anti-CDK4	Bio-Rad	VMA00520
Anti-Rb	ABclonal	A3618
Anti-ATP5A	Proteintech	14676-1-AP
Anti-PGK1	Proteintech	17811-1-AP
Anti-MMP9	Proteintech	10375-2-AP
Anti-SDHA	Proteintech	14865-1-AP
Anti-UQCRC2	Proteintech	14742-1-AP
Anti-β-actin	Proteintech	60008-1-Ig
Anti-E-Cadherin	BD Biosciences	610182
